# When to start: as soon as possible

**DOI:** 10.7448/IAS.15.6.18070

**Published:** 2012-11-11

**Authors:** M Saag

**Affiliations:** 1 University of Alabama, Birmingham, USA

## Abstract

Debate regarding “When to start” antiretroviral (ARV) therapy has raged since the introduction of zidovudine in 1987. Based on the entry criteria for the original Burroughs Wellcome (002) study, the field has been anchored to “CD4 counts” as the prime metric to indicate ARV treatment initiation for asymptomatic HIV-positive individuals. The pendulum has swung back and forth, based mostly on the efficacy and toxicity of available regimens. In today's world, several factors have converged that compel us to initiate therapy as soon as possible: (i) The biology of viral replication (1 to 10 billion viruses/day) screams that we should be starting early. (ii) Resultant inflammation from unchecked replication is associated with earlier onset of multiple co-morbid conditions. (iii) The medications available today are more efficacious and less toxic than in years past. (iv) Clinical trials have demonstrated benefit for all but the highest CD4 strata (>450 to 500 cells/µL). (v) Some cohort studies have demonstrated clear benefit of ARV therapy at any CD4 count, and almost all cohort studies have demonstrated no detrimental effects of early treatment. (vi) In addition to the demonstrated and inferred benefits to the individual patient, we now have a public health benefit of earlier intervention: treatment is prevention. Finally, from a practical/common sense perspective, we are talking about life-long therapy. Whether we start at a CD4 count of 732 or 493/µL, the patient will be on therapy for over 40 to 50 years! There does not seem to be much benefit in waiting, and there is likely to be significant long-term harm. Treat early!

